# Charcot-Marie-Tooth gene, SBF2, associated with taxane-induced peripheral neuropathy in African Americans

**DOI:** 10.18632/oncotarget.12545

**Published:** 2016-10-09

**Authors:** Bryan P. Schneider, Dongbing Lai, Fei Shen, Guanglong Jiang, Milan Radovich, Lang Li, Laura Gardner, Kathy D. Miller, Anne O'Neill, Joseph A. Sparano, Gloria Xue, Tatiana Foroud, George W. Sledge

**Affiliations:** ^1^ Indiana University School of Medicine, Indianapolis, Indiana, USA; ^2^ Dana Farber Cancer Institute, ECOG-ACRIN Biostatistics Center, Boston, Massachusetts, USA; ^3^ Albert Einstein University, Montefiore Medical Center, Bronx, New York, USA; ^4^ Stanford University School of Medicine, Stanford, California, USA

**Keywords:** whole exome sequencing, African American, paclitaxel, peripheral neuropathy, *SBF2*

## Abstract

**Purpose:**

Taxane-induced peripheral neuropathy (TIPN) is one of the most important survivorship issues for cancer patients. African Americans (AA) have previously been shown to have an increased risk for this toxicity. Germline predictive biomarkers were evaluated to help identify *a priori* which patients might be at extraordinarily high risk for this toxicity.

**Experimental design:**

Whole exome sequencing was performed using germline DNA from 213 AA patients who received a standard dose and schedule of paclitaxel in the adjuvant, randomized phase III breast cancer trial, E5103. Cases were defined as those with either grade 3-4 (n=64) or grade 2-4 (n=151) TIPN and were compared to controls (n=62) that were not reported to have experienced TIPN. We retained for analysis rare variants with a minor allele frequency <3% and which were predicted to be deleterious by protein prediction programs. A gene-based, case-control analysis using SKAT was performed to identify genes that harbored an imbalance of deleterious variants associated with increased risk of TIPN.

**Results:**

Five genes had a p-value < 10^−4^ for grade 3-4 TIPN analysis and three genes had a p-value < 10^−4^ for the grade 2-4 TIPN analysis. For the grade 3-4 TIPN analysis, *SET binding factor 2* (*SBF2*) was significantly associated with TIPN (p-value=4.35 x10^−6^). Five variants were predicted to be deleterious in *SBF2*. Inherited mutations in *SBF2* have previously been associated with autosomal recessive, Type 4B2 Charcot-Marie-Tooth (CMT) disease.

**Conclusion:**

Rare variants in *SBF2*, a CMT gene, predict an increased risk of TIPN in AA patients receiving paclitaxel.

## INTRODUCTION

The taxanes are commonly employed chemotherapeutic agents for a variety of malignancies.[1] While improving disease-specific outcomes, they also can impart substantial morbidity. The most common and particularly troublesome toxicity is taxane-induced peripheral neuropathy (TIPN).[2] TIPN can be severe, limit function, cause pain, and may be irreversible. Currently, there are no agents that have been proven to prevent this toxicity and very few have demonstrated benefit in improving symptoms after the toxicity has occurred.[3] Several demographic variables have been shown to increase the risk of TIPN, including: older age, obesity, and the dose and schedule of the taxane.[4] Previously, we found that patients who were genetically defined as African American (AA) had a markedly increased risk for both moderate (grade 2-4) and severe (grade 3-4) TIPN.[5] Despite these findings, much ambiguity remains as to which patients might be at the highest risk for TIPN. Importantly, TIPN is not correlated with superior outcomes in the curative setting.[6] Thus, understanding which AA patients might be at exceptional risk would be beneficial for proper counseling and therapeutic decision-making when considering the risk to benefit ratio for a specific clinical scenario.

Prior correlative studies have been conducted to uncover inherited genetic markers which confer an increased risk for TIPN.[7–15] The majority of these studies have focused on patients of European ancestry (EA) due to the under-representation of AA patients in many clinical studies across the United States. In addition, most studies have focused on the role of common variants, typically those with a minor allele frequency (MAF) greater than 3%. Recent advances in genomic technology has allowed for evaluation of rare coding (exonic) variants at a reasonable speed and price. The rare-variant, common disease hypothesis posits that rare coding variants are more likely to impose a larger effect on risk than common (typically non-exonic) variants.[16, 17] Discovery of variants with large effect size is clinically more beneficial and has greater statistical power when interrogating data sets of modest sample size. In this study we used whole exome sequencing (WES) to test the hypothesis that rare coding variants impact the risk of TIPN for AA patients.

## RESULTS

### Significant covariates for TIPN in African American patients in E5103

**Figure 1:**
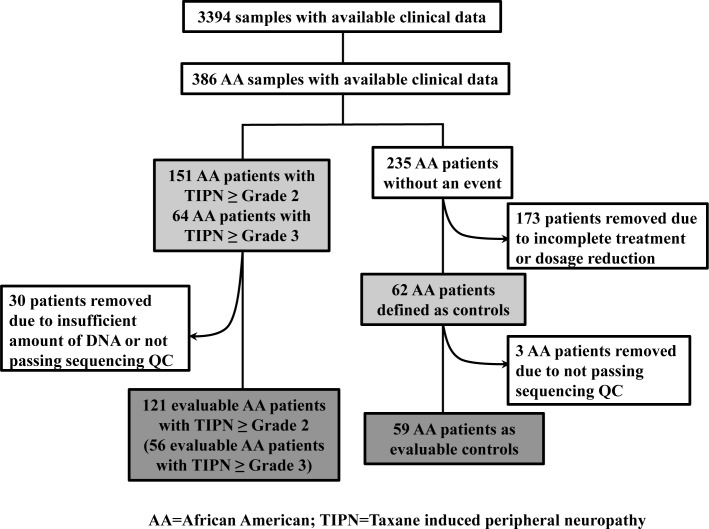
CONSORT diagram for E5103

We previously reported common demographic predictors of TIPN in E5103 and that AAs in the parent trial were markedly more likely to experience both grade 2-4 (HR=2.1; P-value= 5.6 x 10^−16^) and grade 3-4 (HR=2.6; P-value= 1.1 x 10^−11^) TIPN when compared with other races.[5] In the subgroup of previously genome wide genotyped AA patients in E5103, 39.1% experienced grade 2-4 TIPN and 16.6% experienced grade 3-4 TIPN (Figure 1). In this analysis, we evaluated covariates for the population of AAs that underwent WES. Significant covariates in the grade 3-4 TIPN analysis included study arm, PC4 and PC9. Significant covariates in the grade 2-4 TIPN analysis included BSA, arm, PC2 and PC7.

### Top associations for whole exome sequencing in E5103

**Figure 2:**
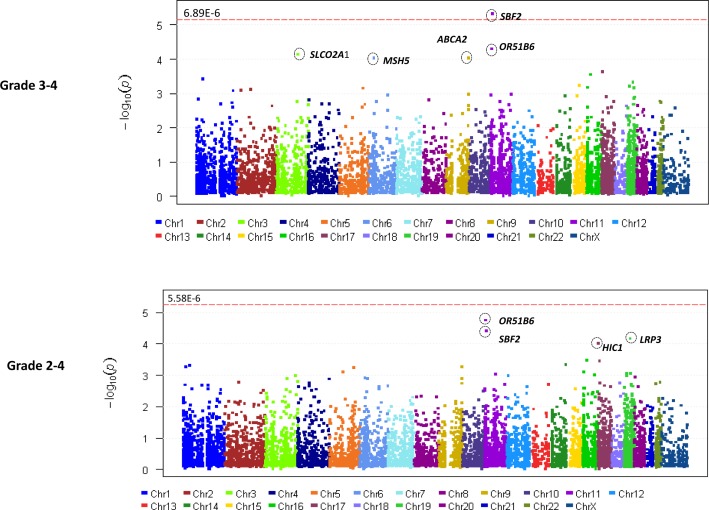
Manhattan plot (**A**) for Grade 3-4 TIPN from AA patients in ECOG-5103. X-axis indicates the chromosomal position of each gene analyzed; Y-axis denotes magnitude of the evidence for association, shown as −log_10_(p-value); Each dot represents an evaluable gene. (**B**) Grade 2-4 TIPN from AA patients in ECOG-5103. X-axis indicates the chromosomal position of each gene analyzed; Y-axis denotes magnitude of the evidence for association, shown as −log_10_(p-value); Each dot represents an evaluable gene. The dashed line indicates the genome-wide significance threshold.

In total, 583,362 variants were identified. After removing variants with missing rate > 20% and those which were monomorphic, 219,607 variants were used for annotation by ANNOVAR. 43,084 rare and predicted to be deleterious, variants were retained for gene-based analyses. 24 samples with missing rates > 30% were excluded in addition to 9 samples which did not have sufficient DNA for sequencing. A total of 180 samples (121 with grade 2-4 TIPN, 56 with grade 3-4 TIPN and 59 controls) passed QC and were evaluable in further analysis (Figure 1). Gene-based analysis required at least 2 variants to be retained in a given gene for it to be included in the analysis. For the grade 3-4 analysis, 7,260 genes had more than 2 variants, thus setting the threshold for significance at p-value < 6.89 x10^−6^. For the Grade 2-4 analysis, 8,958 genes had more than 2 variants, thus setting the threshold for significance at p-value < 5.58 x10^−6^. The results of the gene-based association analyses are shown in Table 1 and Figure 2. Five genes had p-value < 10^−4^ for grade 3-4 TIPN. Three genes had a p-value < 10^−4^ for grade 2-4 TIPN.

**Table 1:** Top genes associated with TIPN in the AA population from E5103

Gene	Grade 3-4 TIPN	Grade 2-4 TIPN
	*P*-value	*P*-value
*SBF2*	4.84E-06	3.94E-05
*OR51B6*	5.08E-05	1.80E-05
*SLCO2A1*	7.37E-05	1.31E-03
*ABCA2*	9.29E-05	5.40E-04
*MSH5*	9.48E-05	1.21E-03
*ZZEF1*	2.33E-04	5.15E-03
*POLR3E*	2.86E-04	3.43E-04
*GNL2*	3.94E-04	4.99E-04
*LRP3*	4.83E-04	6.92E-05
*WDR72*	5.91E-04	2.80E-03
*RGL3*	6.19E-04	8.71E-04
*SEPT12*	6.77E-04	2.58E-03
*CCER2*	6.92E-04	2.00E-03
*SH3RF2*	7.17E-04	5.79E-04
*POLR1A*	7.67E-04	1.74E-03
*C2orf44*	8.20E-04	7.02E-03
*PARP1*	8.41E-04	2.87E-03
*CNTROB*	3.53E-03	3.64E-04
*EXOC3L4*	5.95E-03	4.68E-04
*PLEKHM2*	9.17E-03	5.59E-04
*MTX3*	5.24E-03	7.90E-04
*NMRAL1*	1.26E-02	7.98E-04
*GLIS2*	3.06E-02	8.08E-04
*HAUS5*	2.11E-03	9.02E-04
*PYGM*	4.54E-03	9.45E-04

**Figure 3:**
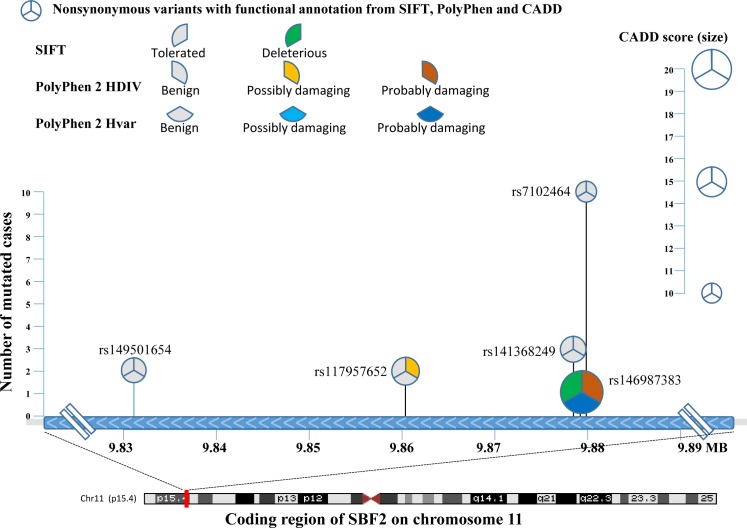
Comprehensive representation of the 5 rare variants detected in the cases from AA patients with grade 3-4 TIPN The X-axis denotes the chromosomal location of each mutation and the Y-axis denotes the number of patients with TIPN that had a given mutation. Each pie-wedge shape & onentation represents the program prediction method and the color represents the type of mutation. The size of the pie represents the CADD score.

**Figure 4:**
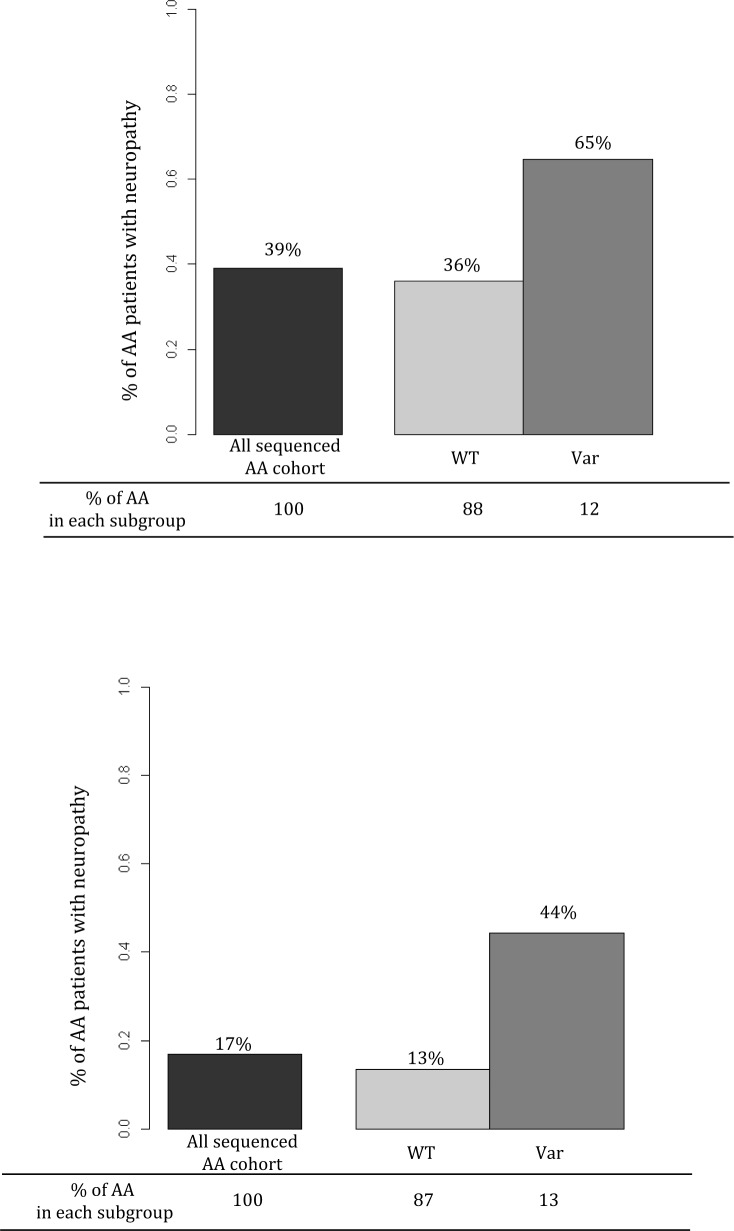
Estimated frequency of TIPN for the entire AA population (black bar), those who have no deleterious mutations in *SBF2* (light gray bar), and those who carry any *SBF2* deleterious mutations (dark gray bar) Each colored bar represents the estimated frequency of TIPN based on the relative likelihood of an event. The percentage value above each bar represents the estimated likelihood of a patient with that variant or genotype experiencing TIPN. The percentage value on the x-axis represents the fraction of the AA population with that specific genotype. Top graph represents grade 2-4 TIPN and bottom graph represents grade 3-4 TIPN.

For grade 3-4 TIPN, the top association was with *SET binding factor 2 (SBF2)* and was statistically significant for an increased risk (p-value=4.35 x10^−6^). Five mutations were predicted to be deleterious in SBF2 (Figure 3 and Table 2). These 5 mutations in *SBF2* were subsequently confirmed using Taqman-based assays. When comparing the estimated frequency of TIPN using the relative likelihood of an event, those patients who carried any of the five deleterious mutations in *SBF2* had a markedly increased risk of TIPN as compared with those who did not carry a variant and had grade 2-4 TIPN (OR=3.26) or grade 3-4 TIPN (OR=5.09); Figure 4. No genes were significantly associated with grade 2-4 TIPN.

**Table 2:** Total counts of each rare variant in *SBF2*

SNP	Grade 3-4 TIPN	Grade 2-4 TIPN	Controls
	(*N*=56)*	(*N*=121)*	(*N*=59)*
*rs149501654*	2	2	0
*rs117957652*	1	2	0
*rs141368249*	2	3	0
*rs146987383*	0	1	0
*rs7102464*	7	10	3

* Number of samples passed QC and were evaluable for final analysis

## DISCUSSION

TIPN is a major complication for many cancer survivors. Unfortunately, there are few identified indicators that can predict whether an individual will develop TIPN. We previously demonstrated that a genetically defined group of AAs in E5103, receiving a commonly used dose and schedule for paclitaxel, had a markedly increased risk of grade 2-4 TIPN (HR=2.1; p=5.6×10^−16^) and grade 3-4 TIPN (HR=2.6; p=1.1×10^−11^) when compared with other races.[5] In the current study, we have employed WES in a subset of the E5103 AA subsample and identified a gene, *SBF2*, with a higher burden of rare deleterious variants among individuals developing grade 3-4 TIPN.

*SBF2* is on chromosome 11p15 and is a member of the myotubularin–related protein family. *SBF2* encodes for a gene that, when mutated, is known to be associated with a subtype of Charcot-Marie-Tooth (CMT), a known hereditary form of polyneuropathy that develops in adolescence, early adulthood, or middle age. CMT is a progressive motor and sensory neuropathy and is the most common form of hereditary neuropathy. A case report previously described that a patient with ovarian cancer who had pre-existing peripheral neuropathy secondary to CMT immediately developed grade 3 sensory and motor neuropathy after a single dose of carboplatin and paclitaxel.[18] Mutations in *SBF2* cause Type 4B CMT, the only type of CMT with an autosomal recessive pattern of inheritance and characterized by axonal degeneration and myelin outfolding.[19]

Genes contributing to CMT have been previously reported to be associated with TIPN.[7, 8] We previously performed a genome wide association study (GWAS) to identify common variants associated with TIPN among AA in E5103.[5] A potential association between *FCAMR* and a decreased risk of grade 2-4 TIPN was observed (Supplemental Figure 1). Baldwin et al, reported their results from a GWAS of CALGB40101 and the top associations were with SNPs in *FDG4, EPHA5*, and *FZD3*; although the first two did not meet genome wide significance.[7] The *FGD4* variant from the CALGB40101 EA discovery set was replicated in a small cohort of AA patients.[7] Both *FGD4* and *SBF2* are known to be associated with subtype 4 CMT.[20] Few prior studies have tested for the effect of rare variants across the exome or genome on TIPN. Beutler et al., performed massively parallel sequencing across 119 EA TIPN patients, focusing on 49 candidate genes felt to be important in the CMT pathway.[8] In that study, several SNPs in *AREGF10* had a modest association with TIPN. In total, these studies provide substantial evidence that genes in the CMT pathway contribute to the risk of TIPN.

There were no statistically significant associations identified in the grade 2-4 TIPN analysis. Other top genes in both analyses (Table 1) included established drug transporters (*SLCO2A1* and *ABCA2*; ranked #3 and #4, respectively), DNA mismatch repair genes (*MSH5* and *PARP1*; ranked #5 and #17, respectively), and a gene encoding for a microtubule binding complex (*HAUS5*; ranked #24).

Strengths of this study include the use of cutting-edge, expansive genomic sequencing in the context of a large trial in an under-represented population of patients who have increased vulnerability to a potentially irreversible therapy-induced toxicity. This correlative study evaluated patients that received a uniform dose and schedule of paclitaxel within the context of a large, randomized, phase III breast cancer clinical trial [21] with rigorous data collection. Another strength of our study was the focus on a genetically defined AA population, a subgroup with a substantially increased risk for TIPN in E5103. Unfortunately many of the large clinical trials across the United States that have captured high-throughput genomic data have substantially under-represented racial minorities [22, 23]. This correlative study represents one of the largest pharmacogenetic studies to date using WES with a focus on genetically determined patients of African descent. This dataset, then, allowed for the direct, rather than the typical inferential application of results, to the AA population. The implementation of massively parallel sequencing across the exome allowed for a comprehensive and unbiased evaluation of rare variants to discover the most important genes associated with TIPN. Thus, the discovery of a gene in a pathway known to have implications in a form of hereditary neuropathy has immediate translational implications.

There are also several weaknesses of this study. First, the study was limited to the exome and therefore could not detect potentially important genetic variants in the non-coding region of the genome. In addition, since only genes having at least two retained variants were included in the analysis, approximately 65% of the genes in the genome were not statistically evaluated in this study. Another limitation of this study was the use of the CTCAE grading scale to define the cases of TIPN. There are data to support that use of patient reported outcomes and more detailed clinical examinations are superior and thus should be considered for future validation biomarker studies.[24] The sample size of this study is modest and is therefore only powered to detect association with genes having multiple variants with large effects on TIPN risk. To rigorously evaluate all genes in the genome for their association with TIPN risk would require a sample many orders of magnitude larger than ECOG-5103 so as to have a sufficient number of AA cases with TIPN and AA controls without evidence of neuropathy.

Understanding which patients might be at increased risk of severe and potentially irreversible toxicities is a critical step toward improved patient counseling and monitoring. When competing drug regimens exist, drugs with different toxicities may be selected on a more personalized basis. Specifically, while clinicians might be most concerned about the rare risk of anthracycline-induced cardiac toxicity for a general population, the risk for a non-taxane based regimens might be more favorable for those at increased risk of severe TIPN.[25] Further, a marker for increased risk might help make a nuanced determination for selection of a taxane (i.e. docetaxel) with a slightly lower risk of TIPN even at the cost of increased risk for competing toxicities. There are also situations where the anticipated benefit from a taxane-based regimen is marginal and a marker of increased risk of TIPN might deter the patient from therapy altogether. Further work to understand the clinical impact of this biomarker on clinician and patient decision-making and implementation are critical next steps. The discovery of predictive genetic biomarkers might also point us in the direction of the underlying biological mechanism for this toxicity and subsequently lead to the therapeutic development for agents designed to either prevent or treat this toxicity. Importantly, risk based biomarkers allow for improved personalization of therapy with the promise of helping patients.

## MATERIALS AND METHODS

### ECOG-5103 Overview

ECOG-5103 was a phase III adjuvant breast cancer trial that randomized 4994 patients with node positive or high-risk node negative breast cancer to intravenous doxorubicin and cyclophosphamide every 2 or 3 weeks (at discretion of treating physician) for four cycles (AC) followed by 12 weeks of weekly paclitaxel (P; 80 mg/m2) alone (Arm A) or to the same chemotherapy with either concurrent bevacizumab (Arm B) or concurrent plus sequential bevacizumab (Arm C)[21].

### Previous genome wide genotyping

Germline DNA from whole blood and companion clinical data were available from 3394 patients. This included 386 patients who were genetically determined to be of African descent (Figure 1). Genome wide SNP arrays (either Illumina HumanOmni1-Quad or Human OmniExpress) were performed in two distinct study subsets as described previously.[5, 26] A principal component analysis (PCA) was performed using Eigenstrat and reference data from 11 HapMap phase III populations to identify clusters using the first two eigenvectors computed using common SNPs from these two arrays. Both arrays were previously used to genotype a set of common samples; discrepancies between these two arrays were negligible[27]. Therefore, we have combined the two data sets to perform PCA. Samples clustering with those of African descent were used in these analyses.[5, 26]

### Case and control definitions

Cases: Two definitions of TIPN were employed. A more stringent criteria limited cases to only those experiencing grade 3-4 TIPN (n=64) (Figure 1) as assessed by the Common Toxicity Criteria Adverse Events (CTCAE) version 3.0. A less stringent TIPN definition included as cases those experiencing grade 2-4 TIPN (n=151) (Figure 1). Under both definitions, cases included patients receiving at least one dose of paclitaxel and the neuropathy event occurred during treatment or within 3 months of the last dose of therapy.

Controls: Controls (n=62) (Figure 1) included patients who met all the following: 1). Received all planned doses of paclitaxel; 2). Had follow-up for at least 3 months after the last dose of drug; 3). Did not meet any of the case definitions as outlined above; and 4). Had either paclitaxel or bevacizumab held or modified for any reason (i.e. disease progression or other toxicity).

### Whole exome sequencing in E5103

Germline DNA samples were quantified by fluorometric methods (Qubit, LifeTechnologies, Eugene, USA). Library preparation was performed according to the manufacturer's guidelines. Exomes were enriched from genomic DNA (50-100 ng) via Ion AmpliSeq™ Exome RDY kits. Libraries were subjected to magnetic bead-based clean up and quantified by qPCR prior to sequencing. Adapters and barcodes were incorporated during library construction, which allowed for two samples to be pooled per Ion PI™ chip. Templates were prepared on Ion Chef™ Systems then sequenced on Ion Proton™ Sequencers (LifeTechnologies) for 520 flows. Typically sequencing runs generated 50-90M reads, yielding on average over 100X coverage of the exome with >90% uniformity. Exomes were aligned to the human genome version GRCh37.3. Variants (SNPs, indels, MNPs) were identified by the Torrent VariantCaller 4.2 software. Libraries on average contained over 50,000 variants. Variants with missing rates > 20% were filtered out of the dataset. Samples with missing rates > 30% were excluded.

ANNOVAR (http://annovar.openbioinformatics.org/en/latest/#reference) was used to annotate location, function and MAF of each variant. To focus on rare variants, only those meeting at least one of the following criteria were retained: 1). MAF ≤ 3% in ESP 6500 (http://evs.gs.washington.edu/EVS/) AA population (primary); 2). MAF ≤ 3% in both 1000G (http://www.1000genomes.org/) EA and AA populations (secondary); 3). MAF ≤ 3% in both ExAc (http://exac.broadinstitute.org/) African or Non-Finnish European populations (tertiary); or 4) novel variants that were reported by any of the reference datasets. Only SNPs defined by RefGene as one of the following were retained: frameshift substitution, nonsynonymous, stop gains, stop loss, or unknown. Retained SNPs were predicted to be deleterious by at least one of the following: 1). SIFT,[28] 2). POLYPHEN2, [29] or 3). CADD (with a score ≥10).[30] The genotypes of the identified deleterious SNPs were confirmed in this dataset with TaqMan SNP assays.

### Statistical analysis

A gene-based case-control analysis (SKAT; http://www.hsph.harvard.edu/skat/) was performed to identify genes associated with an increased risk of TIPN. Study arm, body surface area (BSA), and age were considered as covariates in this analysis. Although these samples were genetically defined as AA based on the common variants on the SNP arrays, we included the first 10 principal components (PC1 to PC10) as covariates in the analysis to adjust for subtle population sub-structures often observed in AA samples.[31] Samples with grade 2 TIPN were sequenced separately; therefore, a batch indicator was also included to adjust potential effect of different experiments. The p-value threshold was < 0.05 for inclusion of covariates in the regression model. Only genes having at least two retained variants were included in the gene-based analysis. The significance threshold for the SKAT analysis was determined by correcting for the number of genes tested in each of the TIPN models. Given the rarity of the individual variants as well as the focus on a gene-based analysis, we do not report individual odds ratios for each variant on TIPN risk.

## SUPPLEMENTARY MATERIAL


