# Supplementary material for: Charcot-Marie-Tooth gene, SBF2, associated with taxane-induced peripheral neuropathy in African Americans
Source: Oncotarget. 2016 Oct 9;7(50):82244–53. doi: 10.18632/oncotarget.12545 (PMC5347688; doi:10.18632/oncotarget.12545)
Supplement: Supplementary file 1 [file oncotarget-07-82244-s001.pdf]

Charcot-Marie-Tooth gene, SBF2, associated with taxane-induced peripheral neuropathy in African Americans

Supplementary Material

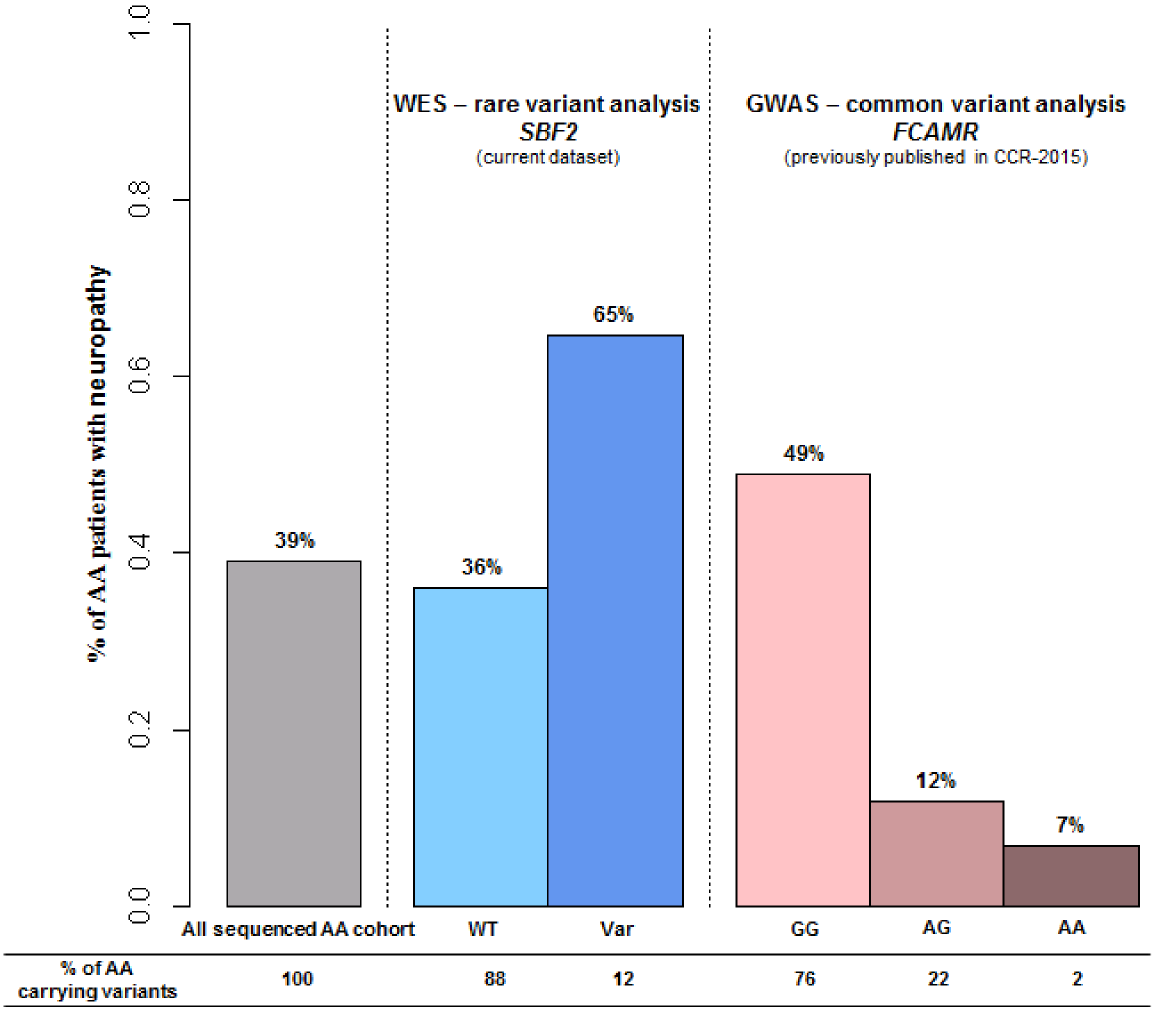

Supplemental Figure 1. Frequency of TIPN Grade 2-4 in AA population broken down by those with and without a mutated SBF2 gene (rare variant; current data) and those who carry one or two variant alleles (*rs1856746*) in *FCAMR* (common variant; based on prior GWAS data).[5] Each colored bar represents the estimated frequency of TIPN based on the relative likelihood of an event. The gray bar represents the frequency of TIPN in the entire E5103 sequenced AA cohort. The blue bars represent an estimated frequency as determined by the odds ratio for an unmutated *SBF2* versus carriage of any deleterious mutation. The pink bars represent the GG, GA, AA genotypes of *rs1856746* in *FCAMR*, respectively. The percentage value above each bar represents the estimated likelihood of a patient with that variant or genotype experiencing TIPN. The percentage value on the x-axis represents the fraction of the AA population with that

specific genotype. WT= wild type gene with no deleterious mutations. Var = variant gene that carries at least one of the 5 deleterious mutations.
